# Novel MicroRNA Biomarkers for Colorectal Cancer Early Diagnosis and 5-Fluorouracil Chemotherapy Resistance but Not Prognosis: A Study from Databases to AI-Assisted Verifications

**DOI:** 10.3390/cancers12020341

**Published:** 2020-02-03

**Authors:** Xueli Zhang, Hong Zhang, Bairong Shen, Xiao-Feng Sun

**Affiliations:** 1 School of Medicine, Institute of Medical Sciences, Örebro University, SE-70182 Örebro, Sweden; zhang.xueli@oru.se (X.Z.); hong.zhang@oru.se (H.Z.); 2 Centre for Systems Biology, Soochow University, Suzhou 215006, China; 3 Department of Oncology and Department of Biomedical and Clinical Sciences, Linköping University, SE-58183 Linköping, Sweden

**Keywords:** miRNA, network models, biomarkers, diagnosis, CRC

## Abstract

Colorectal cancer (CRC) is one of the major causes of cancer death worldwide. In general, early diagnosis for CRC and individual therapy have led to better survival for the cancer patients. Accumulating studies concerning biomarkers have provided positive evidence to improve cancer early diagnosis and better therapy. It is, however, still necessary to further investigate the precise biomarkers for cancer early diagnosis and precision therapy and predicting prognosis. In this study, AI-assisted systems with bioinformatics algorithm integrated with microarray and RNA sequencing (RNA-seq) gene expression (GE) data has been approached to predict microRNA (miRNA) biomarkers for early diagnosis of CRC based on the miRNA-messenger RNA (mRNA) interaction network. The relationships between the predicted miRNA biomarkers and other biological components were further analyzed on biological networks. Bayesian meta-analysis of diagnostic test was utilized to verify the diagnostic value of the miRNA candidate biomarkers and the combined multiple biomarkers. Biological function analysis was performed to detect the relationship of candidate miRNA biomarkers and identified biomarkers in pathways. Text mining was used to analyze the relationships of predicted miRNAs and their target genes with 5-fluorouracil (5-FU). Survival analyses were conducted to evaluate the prognostic values of these miRNAs in CRC. According to the number of miRNAs single regulated mRNAs (NSR) and the number of their regulated transcription factor gene percentage (TFP) on the miRNA-mRNA network, there were 12 promising miRNA biomarkers were selected. There were five potential candidate miRNAs (miRNA-186-5p, miRNA-10b-5, miRNA-30e-5p, miRNA-21 and miRNA-30e) were confirmed as CRC diagnostic biomarkers, and two of them (miRNA-21 and miRNA-30e) were previously reported. Furthermore, the combinations of the five candidate miRNAs biomarkers showed better prediction accuracy for CRC early diagnosis than the single miRNA biomarkers. miRNA-10b-5p and miRNA-30e-5p were associated with the 5-FU therapy resistance by targeting the related genes. These miRNAs biomarkers were not statistically associated with CRC prognosis.

## 1. Introduction 

Colorectal cancer (CRC) is one of the most common types of cancer and its incidence has been increasing during the last decades [1,2], and CRC is also one of the major leading causes of cancer death [1]. In the United States, there are around 145,600 newly-diagnosed CRC patients and 51,020 cancer deaths in 2019, which are estimated by the America Cancer Society [2], and in China, the newly-diagnosed CRC and the cancer deaths are estimated as 521,490 and 245,263, respectively [3]. In the United Kingdom, there are 47,892 new CRC cases and 20,470 patient deaths [4], and in Sweden, the new cases and deaths are 6421 and 3022, respectively [5]. The mortality for the CRC is around 0.35–0.47% of the total diagnosed cases in the world [1,2,3,4,5]. 

It is widely accepted that early diagnosis leads generally to decreasing the cancer mortality significantly [1]. Fecal occult blood test and colonoscopy are currently believed as the most powerful tools to make the early diagnosis for CRC [6]. However, since the fecal occult blood test leads to a rather high false-positive rate it cannot be considered as a specific teat for CRC early diagnosis. The colonoscopy is an invasive examination for the patients with relatively high costs [6]. Accumulating studies have shown that cancer biomarkers such as DNA, RNA and proteins have remarkably provided more accurate evidence for cancer early diagnosis, individual therapy and valuable prediction for cancer prognosis [7,8]. Recently, several small stable molecules, such as microRNAs (miRNA), have been also focused by researchers [9,10]. However, the knowledge concerning these matters is remarkedly lacking. There are still big gaps among the laboratory benches, computer stations and the patients beds. Therefore, it is obviously a great challenge to investigate and develop new strategies for the cancer early diagnosis, more precision therapy and predicting prognosis.

miRNA is a big family of endogenous small stable non-coding RNAs with ~22 nucleotides. The miRNAs play plenty of important roles in the human cellular networks by regulating messenger RNAs (mRNAs) at the post-transcriptional levels [11,12]. Since their stable structure, altered expression and specific detectability, there are several the miRNAs that have been proposed as the biomarkers for the cancer early diagnosis and precision therapy in various types of cancers [9,10].

Our previous study has initially established a comprehensive CRC biomarker database (CBD) based on the published CRC biomarkers [13], in which we collected all reported biomarkers for CRC until 2018 in the PubMed. In the CBD, there are 18 miRNAs that were associated with CRC diagnosis. However, none of these miRNA biomarkers have been further investigated to reach the optimal clinical diagnosis levels. It is, therefore, necessary to further study and clarify the best miRNA biomarkers for the early diagnosis, individual therapy and predicting prognosis in CRC.

During the last decades, the development of computer technology and the big data era, especially the improved machine learning techniques, has provided great opportunities for using bioinformatics techniques in various biomedicine fields which has recently revealed significantly effective ways to discover new biomarkers. Although the bioinformatics models with the classic machine learning methods in gene expression (GE) data have been believed as the golden rule to detect biosignatures, such as the evidence of biomarker predictions [14,15,16], the large heterogeneity among different datasets or/and populations lead certainly to a question to the predicted results and further conclusions.

Biological networks for complex diseases, such as cancers, have been considered as an important research field in bioinformatics [17]. Interestingly, the human social networks have been believed to have similarities with the biological networks: the nodes with similar functions in the networks share similar topological features [18,19]. With this strategy in our minds, we proposed that miRNA biomarkers might have similar topology features in miRNA-mRNA interaction networks. We have developed a software (MiRNA-BD) for miRNA biomarker prediction [20] and it has been used in several different diseases and predicted several useful miRNA biomarkers [21,22,23].

In this study, we integrated different data sources (our CBD and several other public databases) to clarify and predict potential candidate miRNA biomarkers for CRC early diagnosis, and found miRNA-186-5p, miRNA-30e-5p and miRNA-10b-5p as novel potential candidate miRNA biomarkers for CRC diagnosis. The association of the miRNAs with CRC 5-fluorouracil (5-FU) chemotherapy resistance and prognosis was also analyzed.

## 2. Results

**Figure 1:**
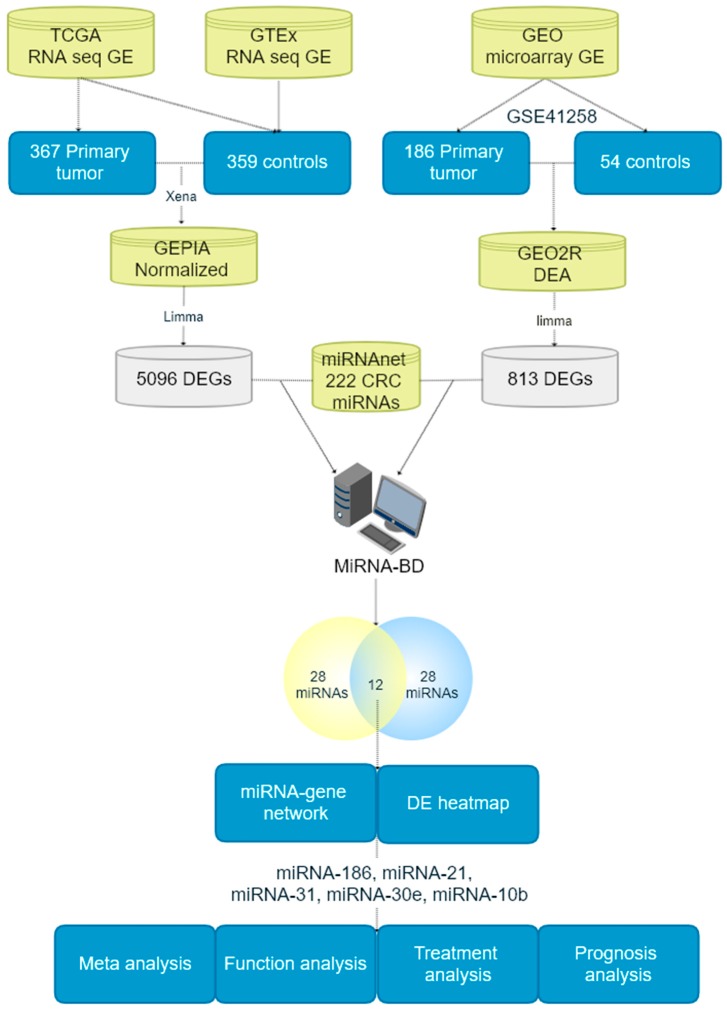
Study pipeline. In this study, a variety of public datasets were utilized to predict novel candidate miRNA biomarkers for colorectal cancer (CRC) early diagnosis via systemic bioinformatics analysis. RNA-seq and microarray gene expression (GE) data concerning CRC patients and normal controls were downloaded from the public databases to make differential expression (DE) analyses (DEA), respectively. The differential expressed genes (DEGs) together with CRC related miRNAs were then input in our MiRNA-BD, a software to predict new biomarkers based on the miRNA-mRNA interaction network. There are 12 promising biomarkers that were predicted by the both RNA-seq and microarray data. Two (miRNA-21-5p and miRNA-31-5p) of them were previously reported as CRC diagnostic miRNA biomarkers. The expression level and miRNA-gene interaction network further identified the novel candidate miRNAs. miRNA-10b-5p, miRNA-30e-5p and miRNA-186-5p were finally found as the novel potential miRNA biomarkers for CRC early diagnosis. These findings were further confirmed by meta-analysis and biological function analysis. Associations of the novel candidate miRNA biomarkers with patients treatment and prognosis were also evaluated.

In this study, we started with differentially expressed (DE) mRNAs from RNA sequencing (RNA-seq) and microarray databases. There were 222 identified CRC associated miRNAs which were collected from miRNet database as the foundations for further topology predictions to construct the CRC specific miRNA-mRNA network. The candidate miRNA biomarkers were further selected by calculating the number of their single regulated mRNAs (NSR) and the number of their regulated transcription factor (TF) gene percentage (TFP). According to the numbers of the NSR and TFP, there were eventually 12 new miRNAs that were found as promising biomarkers for CRC diagnosis. Finally, there were five miRNAs (miRNA-186-5p, miRNA-30e-5p, miRNA-10b-5p, miRNA-21-5p and miRNA-31-5p) were further filtrated as potential candidate miRNA biomarkers from the miRNA-gene interaction network and DE miRNA heatmap. miRNA-186-5p, miRNA-30e-5p and miRNA-10b-5p were three of them newly discovered and confirmed as CRC diagnosis potential miRNA biomarkers, and the other two miRNAs were previously reported. Further, logistic regression based-Bayesian diagnosis meta-analysis and biological functional analysis were performed to verify the findings of the new miRNA biomarkers. The 5-FU therapy resistance and prognosis value of the predicted miRNA biomarkers were also investigated. The pipeline of this study was presented in Figure 1. 

### 2.1. miRNA Biomarker Predictions

In the biomarker prediction model, microarray GE data was collected from GSE 41,258 (186 primary tumor patients and 54 healthy individuals). RNA-seq GE data came from 367 CRC patients and 359 controls. DE analysis was separately conducted for the microarray and RNA-seq data, and There were 5096 and 813 DE genes (DEGs), respectively. The DEGs lists for microarray and RNA-seq data were shown in Table S1. We have drawn the Venn plot to present the overlap between differentially expressed genes from RNA-seq and microarray data in Figure S1. There were 496 overlapping genes that were found in this study. 

Two hundred and twenty two (222) CRC-related miRNAs were collected from the miRNet database, and then input into the MiRNA-BD software together with the DEGs. The 222 CRC related miRNAs were presented in Table S2. Using NSR (NSR ≥ 1, *p* value < 0.05) and TFP (TFP ≥ 1, *p* value < 0.05), 28 predicted miRNA biomarkers were found separately by microarray and RNA-seq data. Table S3 shows the primary prediction results. Table 1 presents the results of the 12 overlapping miRNA biomarkers from the results predicted by microarray and RNA-seq data.

### 2.2. Verifications and Selections for Novel Promising miRNA Biomarkers

**Figure 2:**
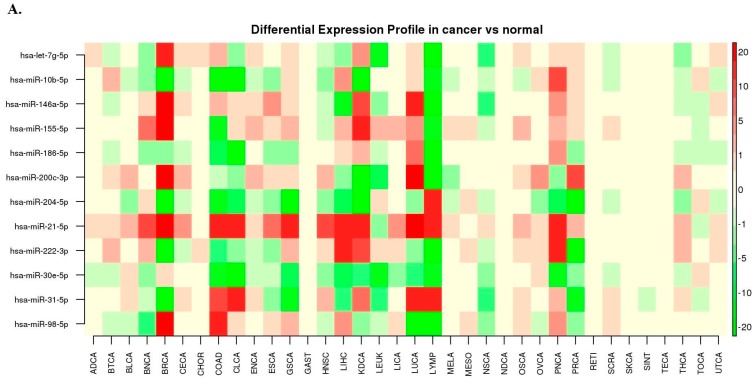

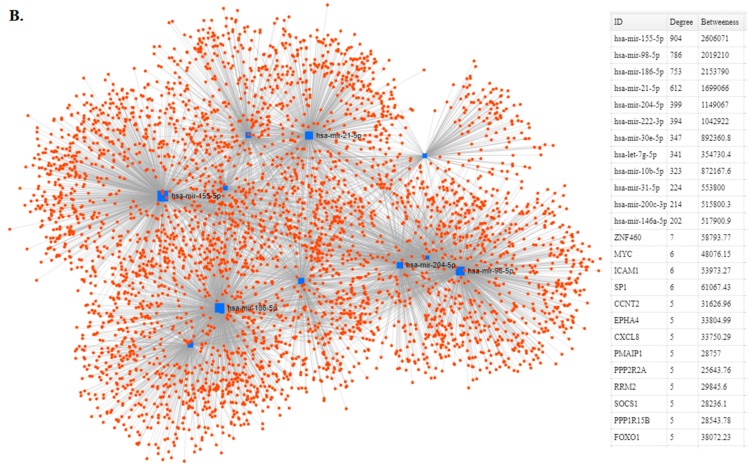

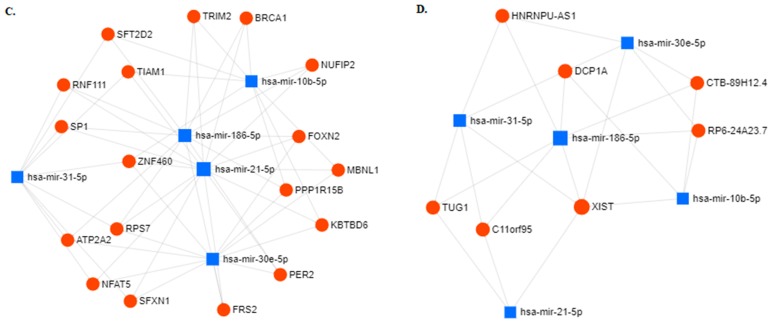

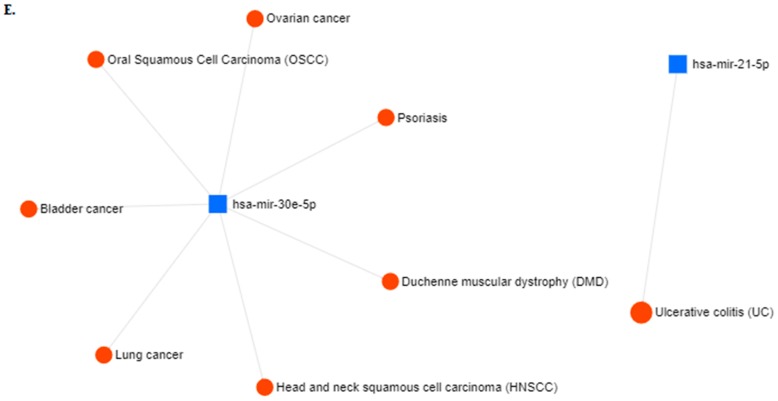

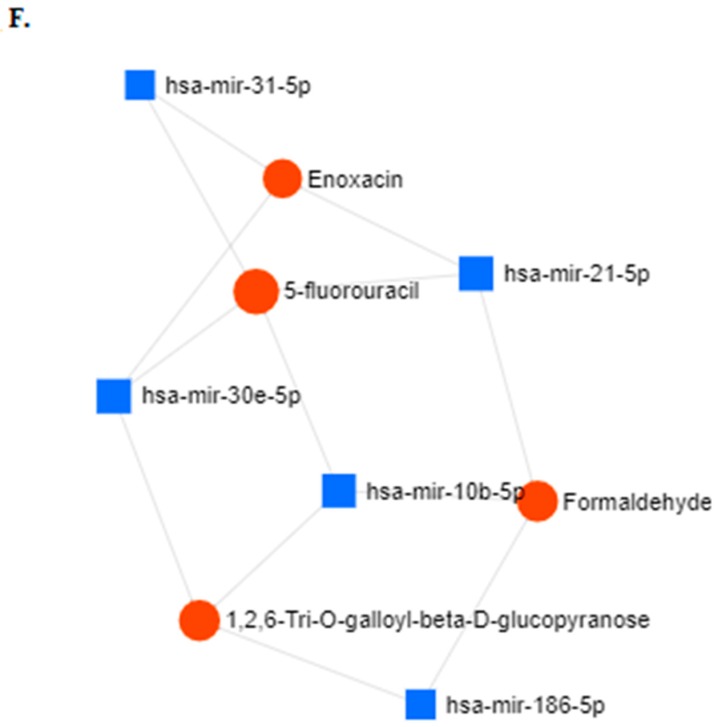
(**A**). Differential expression profiles of the colorectal cancers (CRC) and healthy controls for predicting miRNA biomarkers. miRNA-186-5p, miRNA-30e-5p, miRNA-10b-5p, miRNA-21-5pand miRNA-31-5p were significantly expressed different between the CRC patients and healthy controls. (**B**). miRNA-gene interaction network for the 12 predicted promising miRNA biomarkers. (**C**). miRNA-gene interaction network for miRNA-186-5p, miRNA-30e-5p, miRNA-10b-5p, miRNA-21-5p and miRNA-31-5p. (**D**). miRNA- long non-coding RNA (LncRNA) interaction network for miRNA-186-5p, miRNA-30e-5p, miRNA-10b-5p, miRNA-21-5p and miRNA-31-5p. (**E**). miRNA-disease network for miRNA-186-5p, miRNA-30e-5p, miRNA-10b-5p, miRNA-21-5p and miRNA-31-5p. miRNA-30e-5p were related to many other diseases. (**F**). miRNA-small molecular network for miRNA-186-5p, miRNA-30e-5p, miRNA-10b-5p, miRNA-21-5p and miRNA-31-5p. 5-fluorouracil therapy resistance was associated with miRNA-30e-5p, miRNA-10b-5p, miRNA-21-5p and miRNA-31-5p.

The meta profile heatmap of DE was drawn in order to predict promising miRNA biomarkers, and to observe their DE level in cancers (Figure 2A). miRNA-186-5p, miRNA-21-5p, miRNA-30e-5p, miRNA-10b-5p and miRNA-31-5p were found significant differences between the CRC patients and healthy controls. However, miRNA-21 and miRNA-31 have been found in our CBD database as previously reported CRC diagnosis biomarkers [24,25]. These five miRNAs could be specific diagnosis biomarkers for CRC.

The miRNA-gene interaction network was visualized in Figure 2B, showing that miRNA-186-5p shared similar network topology features with miRNA-21-5p, miRNA-30e-5p, miRNA-10b-5p and miRNA-31-5p were clustered into same level in the miRNA-gene network (similar degree and betweenness).

Taking above evidence together, miRNA-186-5p, miRNA-30e-5p, and miRNA-10b-5p were selected from the DE heatmap and miRNA-gene network as CRC miRNA candidate diagnosis biomarkers. In order to further explore their interaction with miRNA-21-5p and miRNA-31-5p as well as their neighbor genes, the miRNA-gene interaction network for miRNA-186-5p, miRNA-30e-5p, miRNA-10b-5p, miRNA-21-5p and miRNA-31-5p were drawn in Figure 2C. Gene XIST showed a close relationship with all five predicted miRNA biomarkers on the network. Figure 2D–F showed the interaction of these five miRNAs with long non-coding RNAs (LncRNAs), diseases and small molecules. From Figure 2, we found that the three new predicted miRNA biomarkers shared many same related genes, LncRNAs and small molecules with the two identified miRNA biomarkers.

### 2.3. Bayesian Meta-Analysis for Diagnosis 

**Figure 3:**
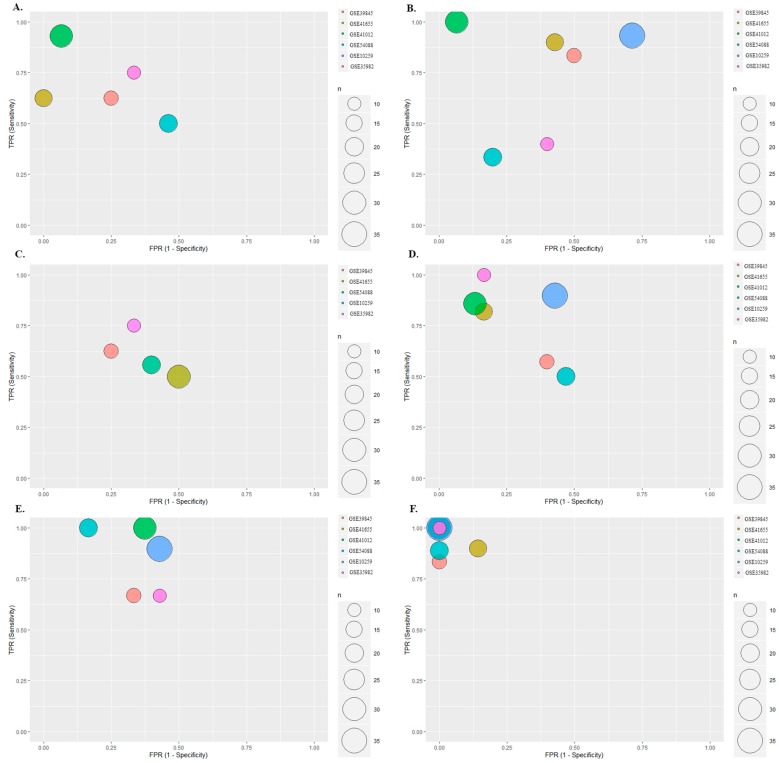
Meta-analyses results for the predicted miRNA biomarkers. Each circle represents specific GSE datasets included in the meta-analysis, and indicates the sensitivity (*y*-axis) and 1-specificity (*x*-axis). The closer to the left corner indicating both sensitivity and specificity close to 1 and the better accuracy of the biomarker for CRC diagnosis). Different sizes and colors of the circles reflect the number of CRC patients for the relevant dataset. (**A**). miRNA-186-5p: miRNA-186-5p played the best role for the diagnosis of patients in GSE41012 and worst in GSE54088; (**B**). miRNA-30e-5p: miRNA-30e-5p showed the highest diagnosis value in the diagnosis of GSE41012 CRC patients; (**C**). miRNA-10b-5p: the patients from GSE35982 and GSE39845 were diagnosed more accurately than the patients in other datasets; (**D**). miRNA-21-5p: miRNA-21-5p performed a diagnostic value in the CRC patients in GSE35982, GSE41012 and GSE41655; (**E**). miRNA-31-5p: miRNA-31-5p as an ideal diagnosis biomarker in the CRC patients from GSE54088; (**F**). Multiple biomarkers showed the best diagnosis value in the CRC patients from all the datasets.

**Figure 4:**
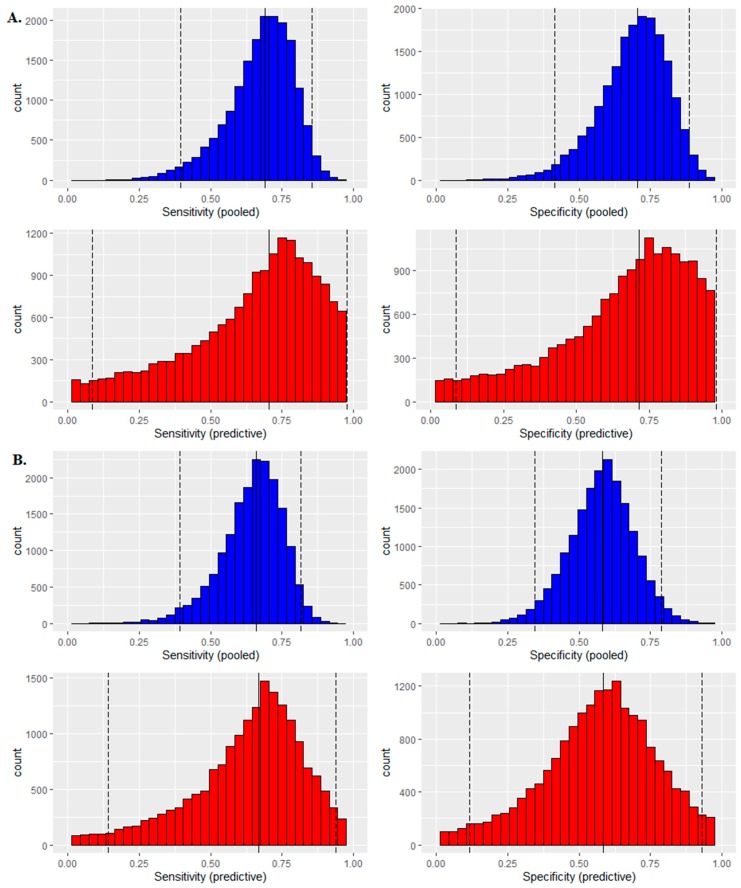

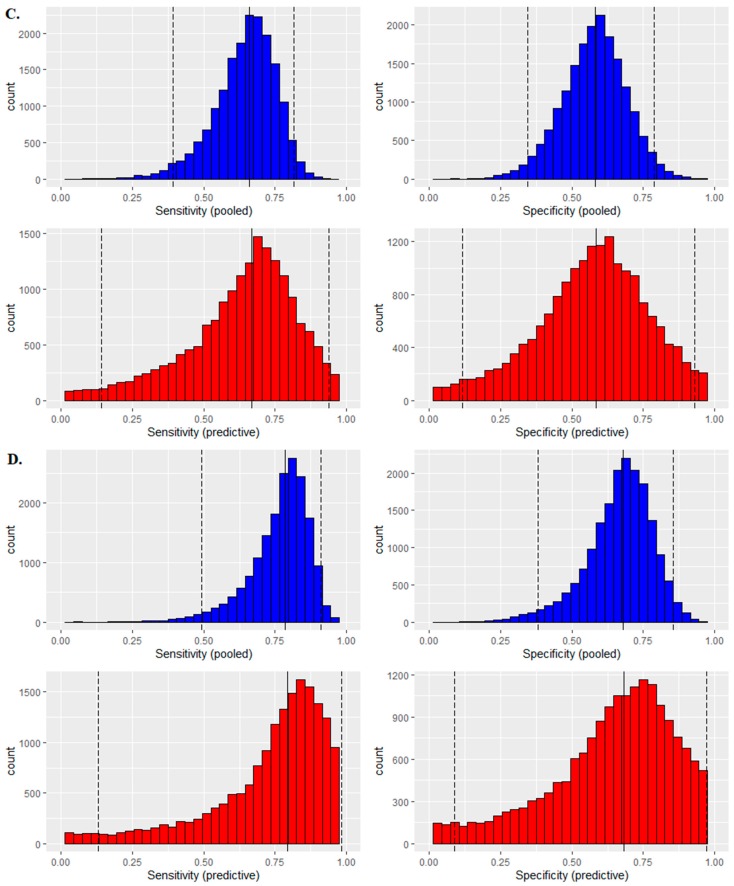

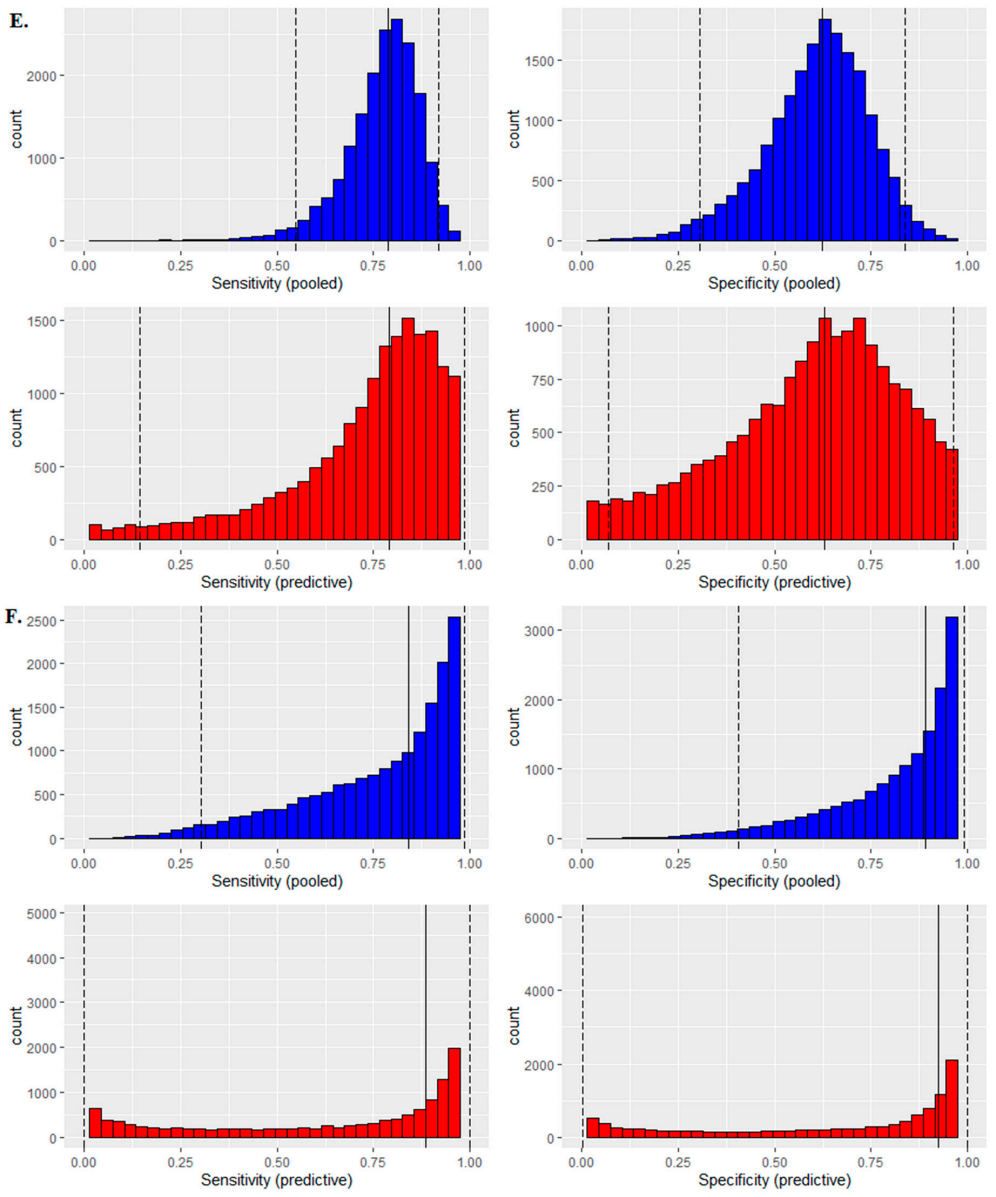
Distribution of pooled sensitivity and specificity, and their predicted posteriors in the Bayesian meta-analyses. (**A**). miRNA-186-5p showed pooled sensitivity: 0.73, prediction sensitivity: 0.74, pooled specificity 0.73 and prediction specificity: 0.74. (**B**). miRNA-30e-5p showed pooled sensitivity: 0.65, prediction sensitivity: 0.66, pooled specificity 0.6 and prediction specificity: 0.61. (**C**). miRNA-10b-5p showed pooled sensitivity: 0.63, prediction sensitivity: 0.64, pooled specificity: 0.6 and prediction specificity: 0.6). (**D**). miRNA-21-5p showed pooled sensitivity: 0.77, prediction sensitivity: 0.78, pooled specificity: 0.72 and prediction specificity: 0.73. (**E**). miRNA-31-5p showed pooled sensitivity: 0.77, prediction sensitivity: 0.77, pooled specificity: 0.63 and prediction specificity: 0.64. (**F**). Analyses of the multiple miRNA biomarkers revealed 0.85 for pooled sensitivity, 0.9 for pooled specificity, 0.89 for prediction sensitivity and 0.93 for the prediction specificity.

In order to further confirm the diagnosis effects of miRNA-186-5p, miRNA-30e-5p and miRNA-10b-5p as novel potential miRNA biomarkers for CRC diagnosis, Bayesian meta-analysis was performed. As a comparison, the meta-analysis for miRNA-21-5p and miRNA-31-5p was performed. Microarray data for 73 early-stage CRC patients and 50 controls were collected from 6 cohorts (GSE3984, GSE41012, GSE41655, GSE54088, GSE10259 and GSE35982) to perform the meta-analysis. The detail amounts of samples were shown in Table S4. Logistic regression was used to transfer the expression data to 2 × 2 table for meta-analysis (Table S4). Figure 3 shows the diagnostic meta-analysis results of the five miRNAs. miRNA-186-5p and miRNA-30e-5p revealed significant diagnostic value in the CRC patients from GSE41012, miRNA-10b-5p and miRNA-21-5p well-performed for CRC diagnosis in GSE 35982, and miRNA-31-5p was predicted as a valuable biomarker in GSE54088. Figure 4 shows the distribution of sensitivity and specificity. All the five miRNAs showed qualified diagnosis accuracy (sensitivity and specificity > 0.6), and miRNA-21-5p (pooled sensitivity: 0.77; prediction sensitivity: 0.78; pooled specificity: 0.72; prediction specificity: 0.73) and miRNA-186-5p (pooled sensitivity: 0.73; prediction sensitivity: 0.74; pooled specificity: 0.73; prediction specificity: 0.74) performed best. Heterogeneity test was used to calculate the batch effects among different studies, and all these meta-analyses showed low batch effects (I^2^ < 25%, Table S4).

### 2.4. Multiple Biomarkers Detections

Multiple biomarkers play generally better roles than single biomarkers. Logistic regression was utilized to combine the novel miRNA candidate biomarkers as multiple biomarkers for CRC diagnosis. Figure 3F and Figure 4F show the Bayesian meta-analysis results for the multiple biomarker combined by miRNA-186-5p, miRNA-30e-5p, miRNA-10b-5p, miRNA-21-5p and miRNA-31-5p, indicating a good performance in the diagnosis of CRC with pooled sensitivity of 0.85, pooled specificity of 0.9, predicted sensitivity of 0.89, and predicted specificity of 0.93.

### 2.5. Biological Function Analyses 

**Figure 5:**
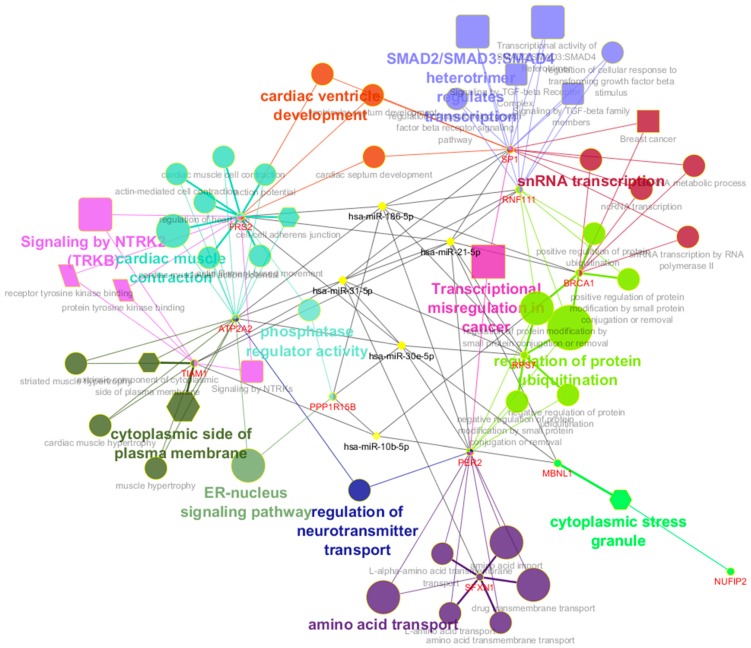
Biological functions of miRNA-186-5p, miRNA-30e-5p and miRNA-10b-5p, miRNA-21-5p and miRNA-31-5p. The different shapes of nodes represent corresponding pathway sources: Ellipse for Gene ontology (GO) in Biological Process, Hexagon for GO in Cellular Component, Octagon for GO in Immune System Process, Parallelogram for GO in Molecular Function, Rectangle for Kyoto Encyclopedia of Genes and Genomes (KEGG) pathways, Round rectangle for Reactoms pathways. The size of pathway nodes represents the significance of enrichment analysis. We set the miRNAs as yellow nodes. The layout and color of other nodes were automatically assigned by Cytoscape.

Biological functions were further analyzed with Kyoto Encyclopedia of Genes and Genomes (KEGG) and Reactome pathway enrichment analysis and Gene Ontology (GO) annotation to explore the associations of miRNA-186-5p, miRNA-30e-5p, miRNA-10b-5p, miRNA-21-5p and miRNA-31-5p with their relevant genes and pathways as shown in Figure 5. The most significant biological pathway from GO in biological process was the regulation of protein ubiquitination, enriched by RNF111, RPS7, PER2 and BRCA1. These genes were regulated and controlled by miRNA-186-5p and miRNA-21-5p. The NTRK2 (TRKB) signaling, the transcriptional activity of SMAD2/SMAD3:SMAD4 heterotrimer, and the SMAD2/SMAD3:SMAD4 heterotrimer regulate transcription were all enriched significantly on Reactoms pathways analysis. The extrinsic component of cytoplasmic side of plasma membrane was the most significant pathway in GO in Cellular Component, and it was mapped by TIAM1 and ATP2A2, which were all regulated by miRNA-31-5p. The most significantly enriched pathways in the GO of molecular functions were the receptor tyrosine kinase binding and the protein tyrosine kinase binding, which were mapped by FRS2 and TIAM1, and they did not share the same miRNAs. Table S5 presented the detail enrichment analysis.

### 2.6. miRNA Biomarkers in CRC Chemotherapy Resistance 

In Figure 2F, we found that the four of these miRNAs (miRNA-21-5p, miRNA-30e-5p, miRNA-10b-5p and miRNA-31-5p) having relationships with 5-FU, a fundamental chemotherapeutic medicine for CRC. Table 2 shows these miRNAs and their associated target genes that affect 5-FU therapy resistance.

### 2.7. miRNA Biomarkers for Prognosis 

**Figure 6:**
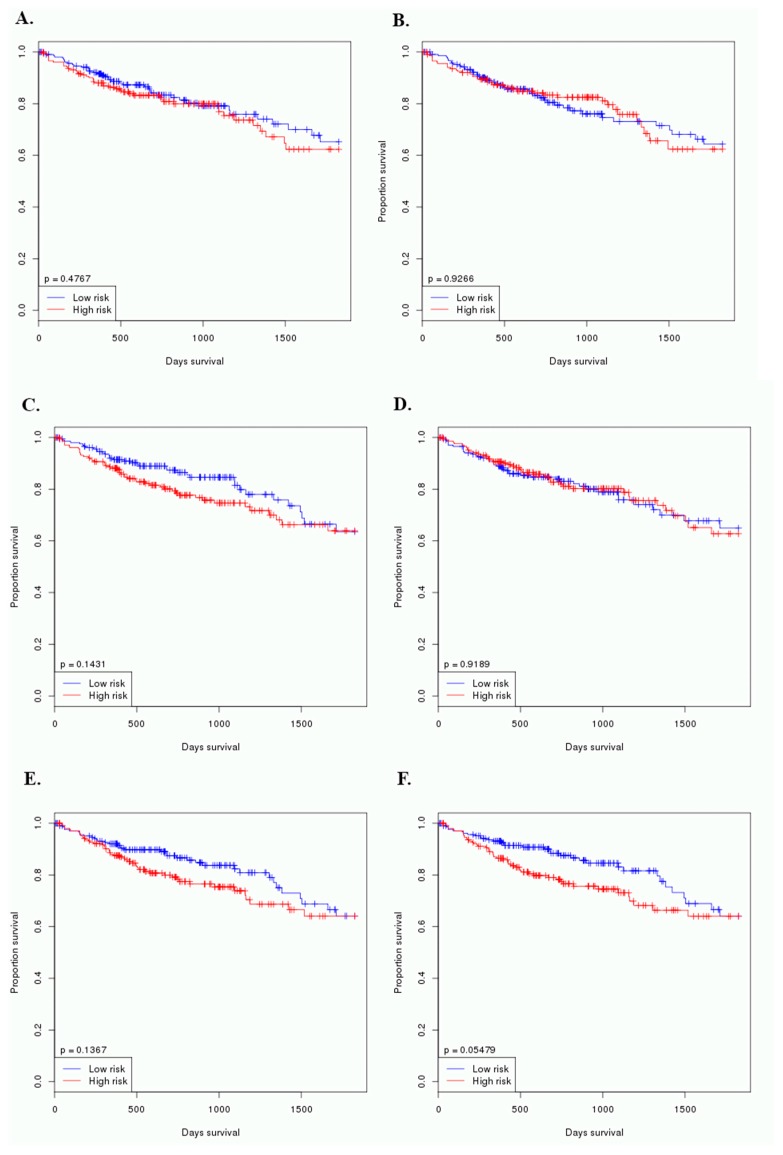
Associations of the predicted miRNA biomarkers and the combinations of the miRNA biomarkers with CRC patient survival. (**A**). miRNA-186-5p; (**B**). miRNA-30e-5p; (**C**). miRNA-10b-5p; (**D**). miRNA-21-5p; (**E**). miRNA-31-5p, and (**F**). combination of the miRNA biomarkers.

In this study, 424 CRC patients were involved in the prognosis test for miRNA candidate biomarkers. The sample sets were arranged as two equal parts according to their survival time, and high/low risks were represented as long-survival/short-survival patients groups. The *p* values were from log-rank tests. Figure 6 showed the CRC patient survival based on the analyses of the miRNAs and their multiple biomarkers, and there were no statistical differences tween the five predicted miRNAs and CRC prognosis (*p* values > 0.05).

## 3. Discussion

We have previously created a CRC biomarker database (CBD) which collected all the reported miRNA biomarkers and their essential biomedicine information for CRC [13]. However, there was no detail information concerning the ideal miRNAs for CRC diagnosis, individual therapy and prognosis [26,27]. It is, therefore, a significant challenge to further evaluate the reported miRNAs and to predict novel candidate miRNAs for early diagnosis, precision therapy and predicting prognosis in CRC. 

There are several public biomarker prediction tools [14,15,16], which are based on the expression of biosignature. However, their outcomes are limited to low robustness due to the heterogeneity of the different populations. In order to improve the limitation and robustness of the miRNA biomarker prediction models and to reduce heterogeneity, we have established a miRNA biomarker prediction software called MiRNA-BD, based on the topology regularity of miRNA-mRNA interaction network [20]. By using the MiRNA-BD, the experiment data from miRTarBase, TarBase, miRecords and miR2Disease and the bioinformatics predicted data from HOCTAR, ExprTargetDB and starBase can be used to construct the miRNA-mRNA network. We have used the MiRNA-BD to predict miRNA biomarkers in prostate cancer [22], pediatric acute myeloid leukemia [28] and CRC with neoadjuvant chemoradiotherapy [29], and found some useful miRNA biomarkers. In these studies, the microarray data was the only resource collected from the Gene Expression Omnibus (GEO) database. During the latest decade, the improvement of sequencing technology has provided better RNA-seq data for bioinformatics analysis. In this study, we used the RNA-seq data from 726 CRC patients and microarray data from 240 CRC patients to construct the model to predict novel miRNA biomarker, respectively, and the overlaps of the predicted miRNA biomarkers were selected as the foundations for further analysis and verification. There were 28 miRNA diagnostic biomarkers predicted from both RNA-seq and microarray datasets, and 12 miRNAs were overlapped as the foundations for further selections and verifications. 

Both the statistical performance and scope of application are needed to be considered under the discovery of the candidate miRNA biomarker. The heatmap of GE for different cancers was used to verify our primary results and further select biomarker candidates. The both 3′ and 5′ miRNAs were used as the foundations for the potential biomarker predictions. According to the primary prediction result in Table 1, the two 3′ miRNAs (miRNA-200c-3p and miRNA-222-3p) were predicted. Since they did not perform well in the differential miRNA expression heatmap (Figure 2A) and their network topology features were not similar to the identified miRNA biomarkers, we excluded them in the final list of the biomarker candidates. There were five miRNAs (miRNA-186-5p, miRNA-10b-5, miRNA-30e-5p, miRNA-21-5p and miRNA-31-5p) were eventually predicted as miRNA candidate biomarkers for CRC early diagnosis since their expression in the CRCs and normal controls were significantly different. After searching in the CBD database, we found that miRNA-21 and miRNA-31 have been previously reported as the diagnostic miRNA biomarkers [24,25]. From our previous studies, CRC biomarkers always showed similar topology features in human biological networks. We found that the three new predicted miRNAs had similar topology features with two proved miRNA biomarkers, which convinced our results further.

Meta-analysis has been playing an essential role in evidence-based medicine since it can summarize the findings from various studies and give statistical conclusions for corresponding topics. Since there are more and more sequencing data that is generated from new sequencing techniques it is worth to use an alternative meta-analysis to analyze the new sequencing data from the findings of these studies. In a previous study, logistic regression has been used to manage the microarray data and to get the needed 2 × 2 table for further the diagnostic meta-analysis. We found that a protein, chromogranin A, could be a promising candidate biomarker for the early diagnosis of CRC [30]. In this study, we continued to use logistic regression to preprocess the miRNA data from CRC GE data and got the 2 × 2 table for diagnostic meta-analysis. The Bayesian meta-analysis, as a new diagnosis meta-analysis, was utilized to verify the diagnosis value of the five predicted candidate miRNA biomarkers, which can provide both the common statistical and predicted parameters. 

Biological network analysis is an important composer in the field of biomarker study, which could give us a complete picture at the molecular levels among the biomarkers and their biological functions. In the previous biomarker studies, we found that the CRC biomarkers shared some general rules in pathways and complex networks. In this study, we followed the similar research strategies and explored the novel candidate miRNA to the most common regulation pathways for CRC miRNA biomarkers to further verify the findings. There were several experimental evidence which supported our findings, such as ubiquitin ligase TRIM65 promoted CRC metastasis by targeting ARHGAP35 for protein degradation (the Regulation of protein ubiquitination) [31], the NTRK2 (TRKB) signaling pathway has been proven as the treatment target for CRC [32], and EGFR on the receptor tyrosine kinase binding pathway associated with patients with chemotherapy-refractory wild-type KRAS exon 2 metastatic colorectal cancer [33]. The interaction networks for the five predicted candidate miRNA biomarkers with genes, LncRNAs, diseases and other small molecules were clearly presented. We realized that several miRNAs related genes and LncRNAs under the discovery of miRNA biomarkers played vital roles in the diagnosis of CRC. The miRNA-30e-5p showed high correlations with several other diseases, indicating that our research strategies might be adapted to other types of cancers and even other diseases. miRNA-21-5p, miRNA-30e-5p, miRNA-31-5p and miRNA-10b-5p were found to have significant relationships with 5-FU therapy resistance in CRC and mechanisms behind the functional genes regulated by these miRNAs in the 5-FU therapy resistance were need to be further investigated. 

The combinations of multiple biomarkers have been proven to have significantly better effects than the single biomarkers for early diagnosis, better therapy and predicting prognosis in CRC [27]. In this study, the effects of various combinations of the five predicted candidate miRNAs on the CRC early diagnosis have been examined, and the multiple miRNAs showed always statistically better predictions than the single miRNAs, indicating that combinations of multiple biomarkers should be considered in the biomarker investigations and the clinical applications. We observed that various single miRNA biomarkers played different roles in CRC early diagnosis, and the combinations of the miRNAs showed consistently better diagnosis values, which indicates that the multiple biomarkers could expand the scopes for CRC patients.

## 4. Materials and Methods

### 4.1. Data Collection and Preparation

Original data concerning RNA-seq GE data of primary tumors and controls from normal tissue of CRC patients as well as healthy populations were collected from The Cancer Genome Atlas (TCGA) database and the GTEx database. Since the number of the controls in TCGA is relatively small, we also used the controls from GTEx to increase the sample size. Xena project (https://xena.ucsc.edu/) was used to standardize the RNA-seq data from both databases [34], and limma package from the R language was used to make DE analysis.

Microarray data from the cohorts for CRC patients and healthy controls were collected from the GEO database, which provided the genes for the construction of the prediction model and the miRNA expression data for logistic regression-based Bayesian meta-analysis. GEO2R platform was used to normalize the collected data and make DE analyses. The datasets containing the early-stage CRC and normal controls were selected for meta-analyses. The CRC related miRNAs were downloaded from the miRNAnet database and the reported CRC miRNA biomarkers were collected from our CBD database.

### 4.2. MiRNA-BD Model

MiRNA-BD is a miRNA biomarker prediction model based on the topology features on the miRNA-mRNA regulatory network [20]. According to our previous findings, miRNA biomarkers shared some common rules on the miRNA-mRNA network and the amounts of their independently regulated genes and TF genes were more prominent than the classic miRNAs. A study model was developed and defined with two parameters: the number of single-line regulation (NSR) and TF gene percentage (TFP), which was utilized to detect the candidate miRNA biomarkers in this study. Wilcoxon signed-rank test was selected to calculate the NSR or TFP of a miRNA as compared to other miRNAs. The *p* value < 0.05 for NSR and TFP was considered as the statistical significance for miRNA biomarkers.

In this study, we started with DE mRNAs from RNA-seq and microarray data. The miRNAs collected from miRNet database and DE mRNAs were mapped on the human miRNA-mRNA network to construct the CRC specific miRNA-mRNA network, and they were combined by the interaction relationships confirmed by experimental and mathematic evidence from popular databases such as miRTarBase, TarBase, miRecords, miR2Disease, HOCTAR, ExprTargetDB and starBase. The candidate miRNA biomarkers were further selected by calculations the NSR and TFP.

### 4.3. Logistic Regression-Based Bayesian Meta-Analysis

A novel comprehensive meta-analysis consisted of logistic regression and the Bayesian test was conducted to detect the diagnosis value for candidate miRNA biomarkers in CRC. The logistic regression was used to initiate the primary diagnostic test for different miRNA candidate biomarkers in different datasets separately, with GE as independent posterior, state of patients (CRC or healthy controls) as dependent posterior. The 2 × 2 table (true positive (TP), false positive (FP), true negative (TN), false negative (FN) from the results of logistic regression analysis was used to perform the Bayesian diagnostic meta-analysis, based on a scale mixtures bivariate random-effects model. The pooled sensitivity and specificity, as well as the prediction sensitivity and specificity from Bayesian meta-analysis were utilized for measuring the diagnostic accuracy of candidate miRNA biomarkers. I^2^ was used to calculate the batch effects (heterogeneity test) among the studies. I^2^ < 25% was considered as low batch effects.

### 4.4. miRNA DE Heatmap, Biological Interaction Networks and Function Analyses

The meta-profiling heatmap of the DE miRNA in different cancers was drawn to observe the expression levels of miRNA promising biomarkers and further select the final candidate biomarkers.

The interaction networks for identified and candidate miRNA biomarkers with genes, LncRNAs, small molecules, and diseases were drawn to verify the predication results and further detect the relationships between miRNA biomarkers and other biological components. In our biological networks, each node represented the corresponding biological component like gene, miRNA, LncRNA, small molecule, as well as various diseases. The connecting edges among different nodes reflected that they had relationships with each other. We used two network topology features (Degree and Betweenness centrality) on the miRNA-gene network to cluster the candidate biomarkers and make a further selection. The degrees in network were the number of edges that connect the target node. The shortest path is the path with minimized edges between two nodes. The betweenness centrality is the number of shortest paths that pass through the target node.

The biological function analysis (GO annotation in biological function/cellular component/immune system process/molecular function (KEGG and Reactome pathway enrichment analysis) were also used to verify, explain the results and find out essential pathways for miRNA biomarkers detection.

### 4.5. Analytical Tools

The meta-profiling heatmap was drawn by the dbDEMC 2.0 database, which includes all the DE miRNA data for 36 cancers from 436 experiments including 45,294 cancer samples [35]. The miRNAnet, Pharmaco miR and PubMed databases provided the information of miRNA related networks for candidate miRNA biomarkers. Logistic regression for miRNA expression data was calculated with SPSS. Bayesian meta-analysis of diagnostic test was implemented by the “bamdit” package on the R language. The heterogeneity test was conducted by the “mada” package on R language. The biological function analysis was processed on the ClueGO and CluePedia Apps of Cytoscape and miRNet database. The survival curves were generated from the ONCOMIR database. P value calculated by the log-rank test was considered as the effect of prognosis value.

## 5. Conclusions

A novel network-based bioinformatics tools (MiRNA-BD) were used to predict miRNA biomarkers for CRC diagnosis, and we found five candidate miRNAs (miRNA-186-5p, miRNA-10b-5p, miRNA-30e-5p, miRNA-31-5p and miRNA-21-5p) which might be used as the early diagnostic biomarkers in CRC, and three (miRNA-186-5p, miRNA-10b-5p and miRNA-30e-5p) of them as novel miRNAs potential biomarkers. The combinations of miRNA-186-5p, miRNA-10b-5p, miRNA-30e-5p, miRNA-31-5p and miRNA-21-5p showed better effects on early diagnosis of CRC. Four miRNAs (miRNA-10b-5p, miRNA-30e-5p, miRNA-31-5p and miRNA-21-5p) and their target genes could be potential biomarkers for evaluating 5-FU therapy resistance. However, the candidate miRNA biomarkers were not significantly associated with CRC prognosis.

## Figures and Tables

**Table 1:** The detail prediction values for 12 initially predicted miRNA biomarkers. The 12 miRNAs were the overlaps of the predictions from RNA-seq data and microarray data. miRNAs were sorted by the P values for NSR in RNA-seq data.

Predicted miRNAs	RNA-Seq Data				Microarray Data			
	NSR	*p* Value for NSR	TFP	*p* Value for TFP	NSR	*p* Value for NSR	TFP	*p* Value for TFP
miRNA-155-5p	22	1.75 × 10^−46^	0.2338	7.41 × 10^−16^	2	2.16 × 10^−19^	0.375	1.39 × 10^−17^
miRNA-30e-5p	17	1.42 × 10^−43^	0.1656	0.015286	8	7.17 × 10^−43^	0.1892	0.012712
miRNA-21-5p	16	6.67 × 10^−43^	0.1915	1.27 × 10^−7^	6	5.74 × 10^−42^	0.2	0.001904
miRNA-98-5p	12	9.58 × 10^−35^	0.1875	8.76 × 10^−7^	1	1.78 × 10^−5^	0.25	1.02 × 10^−8^
miRNA-200c-3p	9	2.31 × 10^−21^	0.1792	5.81 × 10^−5^	5	2.40 × 10^−40^	0.186	0.022552
miRNA-204-5p	9	2.31 × 10^−21^	0.2277	7.17 × 10^−15^	4	2.04 × 10^−35^	0.2121	1.70 × 10^−4^
miRNA-146a-5p	8	3.61 × 10^−17^	0.175	4.72 × 10^−4^	2	2.16 × 10^−19^	0.25	1.02 × 10^−8^
let-7g-5p	7	1.11 × 10^−14^	0.1681	0.006026	2	2.16 × 10^−19^	0.2143	1.15 × 10^−4^
miRNA-10b-5p	6	7.62 × 10^−11^	0.25	2.43 × 10^−17^	1	1.78 × 10^−5^	0.25	1.02 × 10^−8^
miRNA-31-5p	6	7.62 × 10^−11^	0.1667	0.008075	1	1.78 × 10^−5^	0.2	0.001904
miRNA-186-5p	5	1.30 × 10^−6^	0.1667	0.008075	1	1.78 × 10^−5^	0.2	0.001904
miRNA-222-3p	4	0.013019	0.2222	3.09 × 10^−14^	1	1.78 × 10^−5^	0.2857	5.82 × 10^−12^

R: the number of single-line regulation; TFP: transcription factor gene percentage.

**Table 2:** miRNA predicted biomarkers and their target genes for 5-FU therapy resistance.

miRNA	Gene	Source
miRNA-21-5p	MSH2	Pharmaco miR
miRNA-21-5p	BCL2	Pharmaco miR
miRNA-21-5p	PTEN	Pharmaco miR
miRNA-21-5p	Spry2	Pharmaco miR
miRNA-21-5p	PDCD2	Pharmaco miR
miRNA-30e-5p	BCL2	Pharmaco miR
miRNA-10b-5p	BIM	Pharmaco miR
miRNA-31-5p	FIH-1	Pubmed

## Supplementary Materials

The following are available online at https://www.mdpi.com/2072-6694/12/2/341/s1, Table S1: Differential expressional genes list, Table S2: CRC related miRNAs list, Table S3: Primary results for miRNA biomarker prediction by MiRNA-BD, Table S4: Characteristics of datasets in meta-analysis and results in Logistic regression and heterogeneity test, Table S5: The detail results for biological functional analysis, Figure S1: Venn plot for differential expressional genes in microarray data (MDEGs) and RNA-seq data (RDEGs).
