# Supplementary figures and images for: Novel MicroRNA Biomarkers for Colorectal Cancer Early Diagnosis and 5-Fluorouracil Chemotherapy Resistance but Not Prognosis: A Study from Databases to AI-Assisted Verifications
Source: Cancers (Basel). 2020 Feb 3;12(2):341. doi: 10.3390/cancers12020341 (PMC7073235; doi:10.3390/cancers12020341)

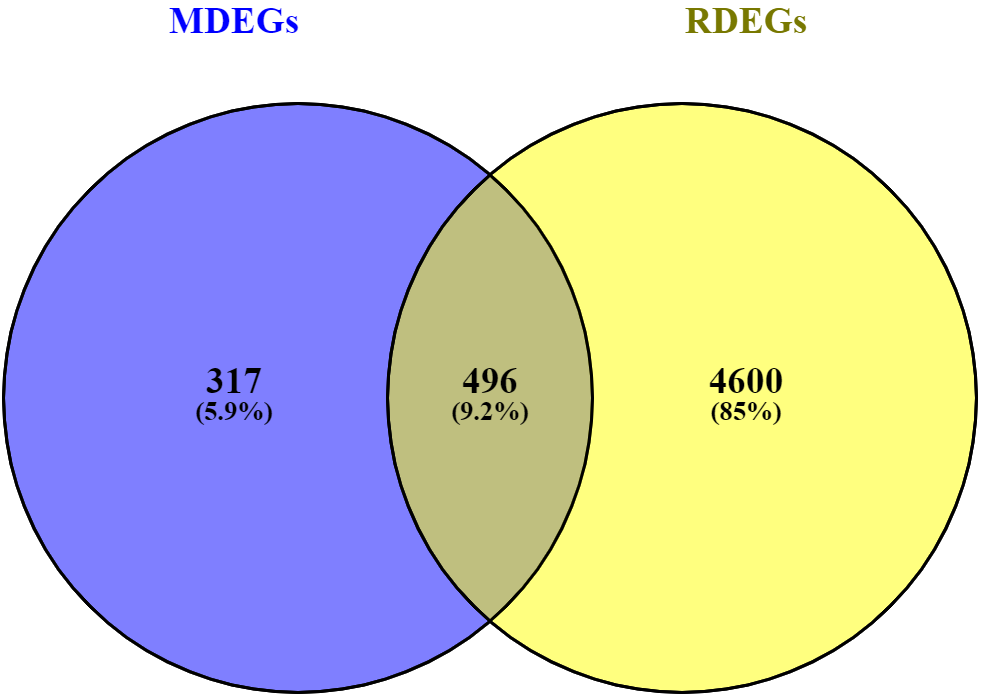

Supplement: Supplementary file 1 [file cancers-12-00341-s001.zip › sm/Figure S1.png]
